# 3-Hydr­oxy-*N*′-(2-hydroxy­benzyl­idene)benzohydrazide

**DOI:** 10.1107/S1600536808027426

**Published:** 2008-09-06

**Authors:** San-Jun Peng, Hai-Yun Hou

**Affiliations:** a College of Chemistry and Biological Engineering, Changsha University of Science and Technology, Changsha 410076, People’s Republic of China; b College of Environmental and Chemical Engineering, Xi’an Polytechnic University, Xi’an 710048, People’s Republic of China

## Abstract

The title compound, C_14_H_12_N_2_O_3_, was synthesized by the condensation of salicylaldehyde with 3-hydroxy­benzo­hydrazide. The dihedral angle between the two benzene rings is 12.4 (2)°. The 2-hydr­oxy group forms an intra­molecular O—H⋯N hydrogen bond with the imide N atom. Mol­ecules are linked through inter­molecular O—H⋯O and N—H⋯O hydrogen bonds into a two-dimensional polymeric structure parallel to the *ab* plane.

## Related literature

For related literature, see: Ali *et al.* (2005 ▶); Eltayeb *et al.* (2008 ▶); Habibi *et al.* (2007 ▶); Jing *et al.* (2006 ▶); Ling *et al.* (2008 ▶); Peng & You (2007 ▶); Peng & Zhou (2007 ▶); Peng, Ping & Song (2007 ▶); Peng, Yang & Zhou (2006 ▶); Peng, Zhou & Yang (2006 ▶); Yehye *et al.* (2008**a* ▶,b* ▶).
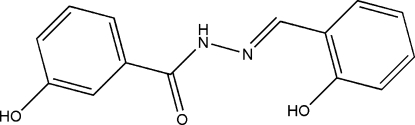


## Experimental

### 

#### Crystal data

C_14_H_12_N_2_O_3_
*M* *_r_* = 256.26
Orthorhombic, 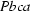

*a* = 14.405 (2) Å
*b* = 9.661 (1) Å
*c* = 17.905 (2) Å
*V* = 2491.8 (5) Å^3^
*Z* = 8
Mo *K*α radiation
μ = 0.10 mm^−1^
*T* = 298 (2) K
0.23 × 0.20 × 0.20 mm

#### Data collection

Bruker SMART 1000 CCD area-detector diffractometer
Absorption correction: multi-scan (*SADABS*; Bruker, 2001 ▶) *T*_min_ = 0.978, *T*_max_ = 0.981
13415 measured reflections
2720 independent reflections
1869 reflections with *I* > 2σ(*I*)
*R*_int_ = 0.039

#### Refinement

*R*[*F* ^2^ > 2σ(*F* ^2^)] = 0.043
*wR*(*F* ^2^) = 0.118
*S* = 1.03
2720 reflections
177 parameters
1 restraint
H atoms treated by a mixture of independent and constrained refinement
Δρ_max_ = 0.13 e Å^−3^
Δρ_min_ = −0.18 e Å^−3^

### 

Data collection: *SMART* (Bruker, 2007 ▶); cell refinement: *SAINT* (Bruker, 2007 ▶); data reduction: *SAINT*; program(s) used to solve structure: *SHELXTL* (Sheldrick, 2008 ▶); program(s) used to refine structure: *SHELXTL*; molecular graphics: *SHELXTL*; software used to prepare material for publication: *SHELXTL*.

## Supplementary Material

Crystal structure: contains datablocks global, I. DOI: 10.1107/S1600536808027426/gk2164sup1.cif

Structure factors: contains datablocks I. DOI: 10.1107/S1600536808027426/gk2164Isup2.hkl

Additional supplementary materials:  crystallographic information; 3D view; checkCIF report

## Figures and Tables

**Table 1:** Hydrogen-bond geometry (Å, °)

*D*—H⋯*A*	*D*—H	H⋯*A*	*D*⋯*A*	*D*—H⋯*A*
O1—H1⋯N1	0.82	1.88	2.6010 (19)	146
O3—H3⋯O2^i^	0.82	1.81	2.5946 (16)	159
N2—H2⋯O3^ii^	0.895 (9)	2.119 (10)	3.0062 (18)	171.0 (18)

Symmetry codes: (i) 
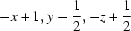
; (ii) 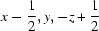.

**Fig. 1.**
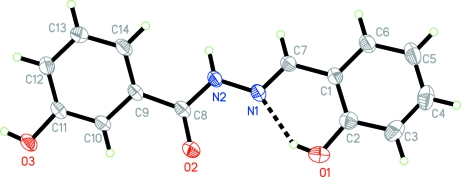
The molecular structure of the title compound, showing the atom-numbering scheme and 30% probability displacement ellipsoids. H atoms are shown as spheres of arbitrary radii.

**Fig. 2.**
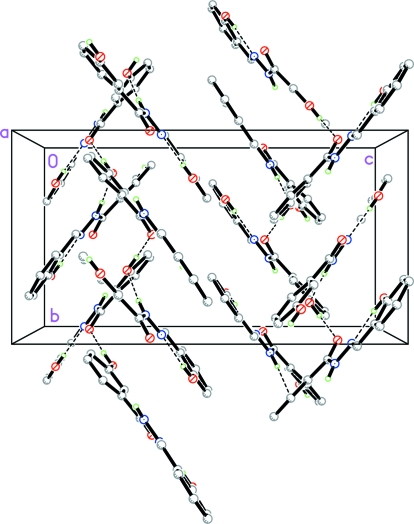
Packing diagram, viewed along the a axis. Hydrogen bonds are shown as dashed lines.

## supplementary crystallographic information

### Comment

Schiff bases derived from the condensation of aldehydes with primary amines play an important role in coordination chemistry (Ali *et al.*, 2005; Eltayeb *et al.*, 2008; Habibi *et al.*, 2007). Recently, we have reported synthesis and crystal structure of some Schiff base complexes (Peng, Yang & Zhou, 2006; Peng, Zhou & Yang, 2006; Peng *et al.*, 2007; Peng & You, 2007; Peng & Zhou, 2007). We report herein the crystal structure of the title compound, Fig. 1.

All the bond lengths are comparable to those observed in other similar compounds (Yehye *et al.*, 2008*a,b*; Jing *et al.*, 2006; Ling *et al.*, 2008). The molecule is not planar and the dihedral angle between the two benzene rings is 12.4 (2)°. There is an intramolecular O–H···N hydrogen bond (Table 1) in each molecule of the compound. The molecules are linked through intermolecular O–H···O and N–H···O hydrogen bonds (Table 1), forming layers parallel to the *ab* plane (Fig. 2).

### Experimental

3-Hydroxybenzohydrazide (0.1 mmol, 15.2 mg) and salicylaldehyde (0.1 mmol, 12.2 mg) were stirred at 318 K in methanol (10 ml) for 30 min. The filtrate was kept open to slowly evaporate for a few days, depositing colorless block-like crystals of the title compound.

### Refinement

The atom H2 attached to N2 was located in a difference Fourier map and refined with N–H distance restrained to 0.90 (1) Å, and with *U*_iso_ set to 0.08 Å^2^. All H atoms bound to carbon and oxygen were refined using riding models with d(C–H) = 0.93 Å, d(O–H) = 0.82 Å, *U*_iso_ = 1.2*U*_eq_(C) and 1.5*U*_eq_(O).

### Crystal data

**Table:**

C_14_H_12_N_2_O_3_	*F*(000) = 1072
*M**_r_* = 256.26	*D*_x_ = 1.366 Mg m^−^^3^
Orthorhombic, *P**b**c**a*	Mo *K*α radiation, λ = 0.71073 Å
Hall symbol: -P 2ac 2ab	Cell parameters from 3092 reflections
*a* = 14.405 (2) Å	θ = 2.7–26.0°
*b* = 9.661 (1) Å	µ = 0.10 mm^−^^1^
*c* = 17.905 (2) Å	*T* = 298 K
*V* = 2491.8 (5) Å^3^	Block, colorless
*Z* = 8	0.23 × 0.20 × 0.20 mm

### Data collection

**Table:**

Bruker SMART 1000 CCD area-detector diffractometer	2720 independent reflections
Radiation source: fine-focus sealed tube	1869 reflections with *I* > 2σ(*I*)
graphite	*R*_int_ = 0.039
ω scans	θ_max_ = 27.0°, θ_min_ = 2.3°
Absorption correction: multi-scan (SADABS; Bruker, 2001)	*h* = −15→18
*T*_min_ = 0.978, *T*_max_ = 0.981	*k* = −11→12
13415 measured reflections	*l* = −22→22

### Refinement

**Table:**

Refinement on *F*^2^	Primary atom site location: structure-invariant direct methods
Least-squares matrix: full	Secondary atom site location: difference Fourier map
*R*[*F*^2^ > 2σ(*F*^2^)] = 0.043	Hydrogen site location: inferred from neighbouring sites
*wR*(*F*^2^) = 0.119	H atoms treated by a mixture of independent and constrained refinement
*S* = 1.03	*w* = 1/[σ^2^(*F*_o_^2^) + (0.0535*P*)^2^ + 0.3997*P*] where *P* = (*F*_o_^2^ + 2*F*_c_^2^)/3
2720 reflections	(Δ/σ)_max_ < 0.001
177 parameters	Δρ_max_ = 0.13 e Å^−^^3^
1 restraint	Δρ_min_ = −0.18 e Å^−^^3^

### Special details

**Table:**

Geometry. All e.s.d.'s (except the e.s.d. in the dihedral angle between two l.s. planes) are estimated using the full covariance matrix. The cell e.s.d.'s are taken into account individually in the estimation of e.s.d.'s in distances, angles and torsion angles; correlations between e.s.d.'s in cell parameters are only used when they are defined by crystal symmetry. An approximate (isotropic) treatment of cell e.s.d.'s is used for estimating e.s.d.'s involving l.s. planes.
Refinement. Refinement of *F*^2^ against ALL reflections. The weighted *R*-factor *wR* and goodness of fit *S* are based on *F*^2^, conventional *R*-factors *R* are based on *F*, with *F* set to zero for negative *F*^2^. The threshold expression of *F*^2^ > σ(*F*^2^) is used only for calculating *R*-factors(gt) *etc*. and is not relevant to the choice of reflections for refinement. *R*-factors based on *F*^2^ are statistically about twice as large as those based on *F*, and *R*- factors based on ALL data will be even larger.

### Fractional atomic coordinates and isotropic or equivalent isotropic displacement parameters (Å^2^)

**Table:**

	*x*	*y*	*z*	*U*_iso_*/*U*_eq_	
O1	0.16163 (8)	1.20579 (14)	0.04590 (8)	0.0677 (4)	
H1	0.1829	1.1427	0.0713	0.102*	
O2	0.33625 (7)	0.98665 (12)	0.15618 (6)	0.0524 (3)	
O3	0.56381 (7)	0.66767 (12)	0.27314 (7)	0.0548 (3)	
H3	0.5826	0.6036	0.2991	0.082*	
N1	0.15527 (8)	0.98711 (13)	0.13048 (7)	0.0419 (3)	
N2	0.19669 (8)	0.88568 (13)	0.17276 (8)	0.0436 (3)	
C1	0.01929 (10)	1.09152 (16)	0.08195 (8)	0.0409 (4)	
C2	0.06764 (12)	1.19810 (18)	0.04586 (9)	0.0493 (4)	
C3	0.01833 (15)	1.3000 (2)	0.00834 (11)	0.0668 (5)	
H3A	0.0501	1.3716	−0.0152	0.080*	
C4	−0.07686 (16)	1.2964 (2)	0.00557 (11)	0.0709 (6)	
H4	−0.1089	1.3653	−0.0200	0.085*	
C5	−0.12534 (13)	1.1922 (2)	0.04022 (11)	0.0663 (6)	
H5	−0.1898	1.1900	0.0380	0.080*	
C6	−0.07735 (11)	1.0909 (2)	0.07839 (9)	0.0533 (4)	
H6	−0.1102	1.0207	0.1022	0.064*	
C7	0.06694 (10)	0.98419 (16)	0.12381 (9)	0.0423 (4)	
H7	0.0331	0.9131	0.1458	0.051*	
C8	0.28882 (10)	0.89504 (16)	0.18561 (8)	0.0411 (4)	
C9	0.32990 (9)	0.78911 (16)	0.23665 (9)	0.0396 (4)	
C10	0.42602 (10)	0.77388 (16)	0.23447 (9)	0.0402 (4)	
H10	0.4612	0.8302	0.2033	0.048*	
C11	0.46926 (9)	0.67575 (16)	0.27832 (9)	0.0407 (4)	
C12	0.41777 (11)	0.59119 (18)	0.32492 (9)	0.0474 (4)	
H12	0.4468	0.5240	0.3538	0.057*	
C13	0.32267 (11)	0.6079 (2)	0.32798 (10)	0.0561 (5)	
H13	0.2878	0.5519	0.3596	0.067*	
C14	0.27860 (11)	0.70631 (19)	0.28484 (9)	0.0517 (4)	
H14	0.2146	0.7172	0.2880	0.062*	
H2	0.1622 (12)	0.8175 (16)	0.1921 (11)	0.080*	

### Atomic displacement parameters (Å^2^)

**Table:**

	*U*^11^	*U*^22^	*U*^33^	*U*^12^	*U*^13^	*U*^23^
O1	0.0516 (7)	0.0702 (10)	0.0815 (10)	−0.0133 (6)	0.0067 (6)	0.0162 (7)
O2	0.0375 (6)	0.0518 (7)	0.0680 (8)	−0.0079 (5)	−0.0066 (5)	0.0050 (6)
O3	0.0286 (5)	0.0524 (7)	0.0835 (9)	0.0054 (5)	−0.0026 (5)	0.0072 (6)
N1	0.0331 (6)	0.0436 (8)	0.0488 (7)	0.0030 (5)	−0.0044 (5)	−0.0028 (6)
N2	0.0297 (6)	0.0423 (8)	0.0586 (8)	0.0007 (5)	−0.0060 (6)	0.0025 (6)
C1	0.0378 (8)	0.0462 (9)	0.0387 (8)	0.0039 (7)	−0.0007 (6)	−0.0018 (7)
C2	0.0507 (10)	0.0523 (10)	0.0448 (9)	−0.0009 (8)	0.0011 (7)	−0.0004 (8)
C3	0.0846 (15)	0.0592 (12)	0.0566 (11)	0.0036 (10)	0.0013 (10)	0.0144 (9)
C4	0.0794 (14)	0.0749 (14)	0.0583 (12)	0.0259 (11)	−0.0111 (11)	0.0105 (10)
C5	0.0491 (10)	0.0885 (15)	0.0614 (12)	0.0206 (10)	−0.0073 (9)	0.0031 (11)
C6	0.0401 (9)	0.0672 (12)	0.0525 (10)	0.0064 (8)	−0.0014 (7)	0.0054 (9)
C7	0.0359 (8)	0.0434 (9)	0.0476 (9)	−0.0005 (6)	0.0000 (7)	0.0001 (7)
C8	0.0312 (7)	0.0424 (9)	0.0497 (9)	−0.0003 (7)	−0.0027 (6)	−0.0081 (7)
C9	0.0286 (7)	0.0440 (9)	0.0461 (8)	−0.0005 (6)	−0.0040 (6)	−0.0075 (7)
C10	0.0296 (7)	0.0395 (8)	0.0516 (9)	−0.0022 (6)	0.0002 (6)	−0.0050 (7)
C11	0.0275 (7)	0.0417 (8)	0.0529 (9)	0.0026 (6)	−0.0050 (7)	−0.0113 (7)
C12	0.0414 (8)	0.0540 (10)	0.0467 (9)	0.0054 (7)	−0.0046 (7)	0.0004 (8)
C13	0.0394 (9)	0.0740 (13)	0.0548 (10)	−0.0012 (8)	0.0048 (7)	0.0130 (9)
C14	0.0282 (7)	0.0729 (12)	0.0541 (10)	0.0016 (7)	0.0006 (7)	0.0026 (9)

### Geometric parameters (Å, °)

**Table:**

O1—C2	1.356 (2)	C4—H4	0.9300
O1—H1	0.8200	C5—C6	1.380 (3)
O2—C8	1.2360 (18)	C5—H5	0.9300
O3—C11	1.3673 (17)	C6—H6	0.9300
O3—H3	0.8200	C7—H7	0.9300
N1—C7	1.2784 (18)	C8—C9	1.494 (2)
N1—N2	1.3745 (18)	C9—C14	1.389 (2)
N2—C8	1.3499 (18)	C9—C10	1.3928 (19)
N2—H2	0.895 (9)	C10—C11	1.379 (2)
C1—C6	1.394 (2)	C10—H10	0.9300
C1—C2	1.401 (2)	C11—C12	1.383 (2)
C1—C7	1.452 (2)	C12—C13	1.380 (2)
C2—C3	1.388 (2)	C12—H12	0.9300
C3—C4	1.373 (3)	C13—C14	1.380 (2)
C3—H3A	0.9300	C13—H13	0.9300
C4—C5	1.373 (3)	C14—H14	0.9300
			
C2—O1—H1	109.5	N1—C7—C1	120.20 (14)
C11—O3—H3	109.5	N1—C7—H7	119.9
C7—N1—N2	117.88 (13)	C1—C7—H7	119.9
C8—N2—N1	118.21 (13)	O2—C8—N2	121.25 (14)
C8—N2—H2	122.0 (13)	O2—C8—C9	122.15 (13)
N1—N2—H2	119.8 (13)	N2—C8—C9	116.60 (13)
C6—C1—C2	118.60 (15)	C14—C9—C10	119.03 (14)
C6—C1—C7	119.53 (15)	C14—C9—C8	124.29 (13)
C2—C1—C7	121.86 (14)	C10—C9—C8	116.68 (13)
O1—C2—C3	118.19 (16)	C11—C10—C9	120.37 (14)
O1—C2—C1	122.45 (15)	C11—C10—H10	119.8
C3—C2—C1	119.36 (17)	C9—C10—H10	119.8
C4—C3—C2	120.71 (19)	O3—C11—C10	116.77 (14)
C4—C3—H3A	119.6	O3—C11—C12	122.77 (14)
C2—C3—H3A	119.6	C10—C11—C12	120.46 (13)
C3—C4—C5	120.68 (18)	C13—C12—C11	119.15 (15)
C3—C4—H4	119.7	C13—C12—H12	120.4
C5—C4—H4	119.7	C11—C12—H12	120.4
C4—C5—C6	119.29 (18)	C14—C13—C12	120.98 (16)
C4—C5—H5	120.4	C14—C13—H13	119.5
C6—C5—H5	120.4	C12—C13—H13	119.5
C5—C6—C1	121.34 (18)	C13—C14—C9	119.98 (14)
C5—C6—H6	119.3	C13—C14—H14	120.0
C1—C6—H6	119.3	C9—C14—H14	120.0

### Hydrogen-bond geometry (Å, °)

**Table:**

*D*—H···*A*	*D*—H	H···*A*	*D*···*A*	*D*—H···*A*
O1—H1···N1	0.82	1.88	2.6010 (19)	146.
O3—H3···O2^i^	0.82	1.81	2.5946 (16)	159.
N2—H2···O3^ii^	0.90 (1)	2.12 (1)	3.0062 (18)	171.(2)

Symmetry codes: (i) −*x*+1, *y*−1/2, −*z*+1/2; (ii) *x*−1/2, *y*, −*z*+1/2.
